# Acute massive gastric dilatation with necrosis and portal vein gas accumulation—a case report

**DOI:** 10.3389/fmed.2026.1796420

**Published:** 2026-06-19

**Authors:** Xiaoxu He, Jun Wang, Yongjiang Zhao, Shumin Ma, Zhiping Wang, Bo Chen, Yongliang Yao

**Affiliations:** Qujing Second People’s Hospital, Qujing, China

**Keywords:** acute gastric dilatation, exploratory laparotomy, gastric necrosis, intestinal obstruction, portal vein gas

## Abstract

Acute gastric distension (AGD) is a rare but life-threatening acute abdominal condition. Despite the rich blood supply to the stomach, extreme distension can lead to ischemic necrosis, perforation, and gas accumulation in the portal vein, with a mortality rate as high as 80–100%. We report the case of a 15-year-old male patient who presented with severe abdominal pain, bloating, and vomiting following a binge-eating episode. Physical examination revealed signs of diffuse peritonitis and shock (heart rate 154 beats per minute). Laboratory results showed severe metabolic acidosis (pH 7.265) and hyperlactatemia (11.3 mmol/L). An abdominal CT scan confirmed massive gastric distension and gas accumulation in the portal vein, biliary tract, and esophageal wall. The patient underwent emergency laparotomy within 2 hours of admission. Intraoperatively, the stomach was found to be distended into the pelvic cavity, with two areas of hemorrhagic necrosis: one on the lesser curvature (4 cm × 5 cm) and another on the posterior wall of the greater curvature (3 cm × 4 cm). Gastric decompression, debridement of the necrotic areas, and creation of a gastrostomy were performed. After 5 days of intensive care unit (ICU) monitoring (for treatment of septic shock), the patient recovered well and was discharged on the 14th postoperative day; follow-up revealed no recurrence. This case demonstrates that even healthy individuals without eating disorders may develop AGD due to acute binge eating. Portal air is a critical warning sign of mucosal necrosis. Early diagnosis via CT and prompt surgical decompression are key to saving the lives of patients with ischemia and shock.

## Introduction

Acute gastric dilatation (AGD) is a rare clinical emergency characterized by rapid filling of the stomach with a large volume of contents within a short period. Due to the severity of this condition, precise incidence statistics are currently unavailable; however, reports indicate that mortality rates from gastric ischemia and perforation caused by acute dilatation can reach 80 to 100% (1, 2). The causes of acute gastric distension remain unclear; they are generally classified as mechanical or non-mechanical. Mechanical causes include pyloric obstruction, while non-mechanical causes include bulimia nervosa or anorexia nervosa. A clear diagnosis can be made for the vast majority of patients through abdominal CT scans (1, 3). Although the stomach exhibits strong tolerance to ischemia due to its rich collateral circulation, extreme and persistent distension can impair blood supply to the gastric wall, leading to gastric ischemia and even transmural necrosis. Once this pathological process occurs, the condition deteriorates rapidly, often accompanied by perforation, septic shock, and multiple organ failure, with an extremely high mortality rate. The presence of gas in the portal vein indicates intra-abdominal hypertension, typically signifying severe necrosis of the gastrointestinal mucosa. Gas-producing bacteria invade the gastric wall and proliferate within the portal venous system, causing mortality rates to increase exponentially (3). We report a case of severe gastric distension caused by binge eating, complicated by localized gastric necrosis and gas accumulation in the portal vein, in which the patient survived emergency exploratory laparotomy, with the aim of raising clinicians’ awareness of this condition.

## Case report

A 15-year-old male patient presented to our hospital in the early hours of the morning following a large hotpot meal, complaining of generalized abdominal pain, bloating, nausea, and vomiting. Vomit containing food particles with a sour, foul odor. There is no blood or coffee-ground-like material in the vomit. Upon admission, the patient presented with severe generalized abdominal pain. Vital signs: T 37.0 °C, P:154 bpm, RR 24 bpm, BP 145/105 mmHg, SpO2 93%. Physical examination revealed abdominal muscle rigidity, marked tenderness, rebound tenderness, and significant abdominal distension. The patient also experienced nausea and vomiting, with vomitus appearing as coffee-ground-like fluid. The patient’s parents divorced when the patient was 2 years old, and the patient has lived with the father since childhood. There is no history of clearly diagnosed psychiatric disorders, constipation, or picky eating.

**Figure 1:**
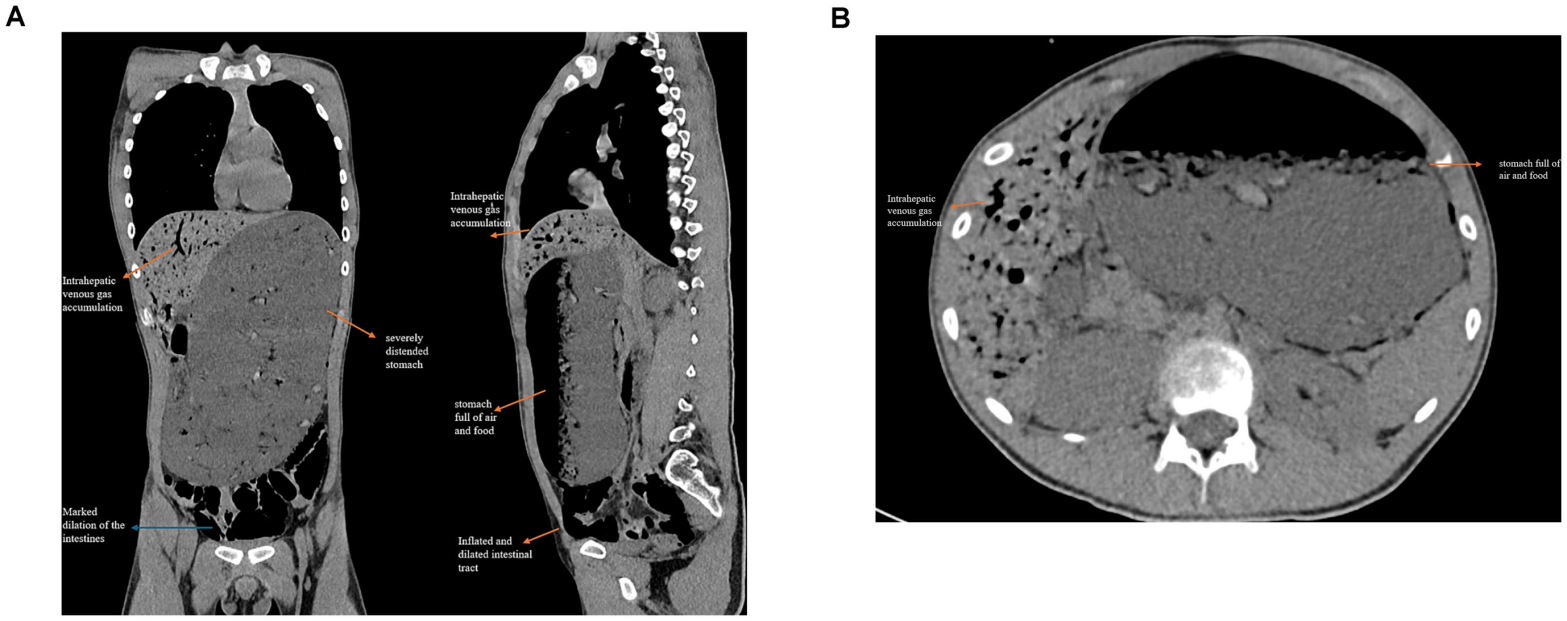
**(A,B)** The CT image shows a significant dilation of the stomach, with gas accumulation in the esophagus, intestines, and portal vein.

Abdominal CT scan reveals severe gastric dilatation, small bowel obstruction, and gas accumulation within the small bowel wall, portal vein, biliary tract, and esophagus, suggesting possible small bowel necrosis (Figures 1A,B). Laboratory test results are shown in the Table 1.

**Table 1:** Emergency laboratory investigations upon admission.

Category	Parameter	Result	Unit	Reference range
Arterial blood gas	pH	**7.265**	–	7.350–7.450
(FiO₂: 33%)	HCO₃^−^	**15.9**	mmol/L	22.0–26.0
	Standard Bicarbonate (SBC)	**16.7**	mmol/L	22.0–27.0
	Actual Base Excess (ABE)	**−10.1**	mmol/L	−3.0 − +3.0
	Standard Base Excess (SBE)	**−11.1**	mmol/L	−3.0 − +3.0
	Lactate	**11.3**	mmol/L	0.5–2.2
	Potassium (K^+^)	3.8	mmol/L	3.5–5.5
Hematology	White Blood Cell (WBC)	**18.82**	×10^9^/L	4.00–10.00
	Neutrophil Count	**17.33**	×10^9^/L	1.80–6.30
	Red Blood Cell (RBC)	5.80	×10^12^/L	4.30–5.80
	Hemoglobin (Hb)	173	g/L	130–175
Cardiac markers	Myoglobin	22.0	ng/mL	0–70.0
	CK-MB	1.23	ng/mL	0–5.0
	hs-Troponin I	0.016	ng/mL	0–0.04
Inflammatory marker	Procalcitonin (PCT)	0.05	ng/mL	<0.5
Organ function	Liver function	Normal	–	–
	Kidney Function	Normal	–	–

Abnormal values are highlighted in bold.

**Figure 2:**
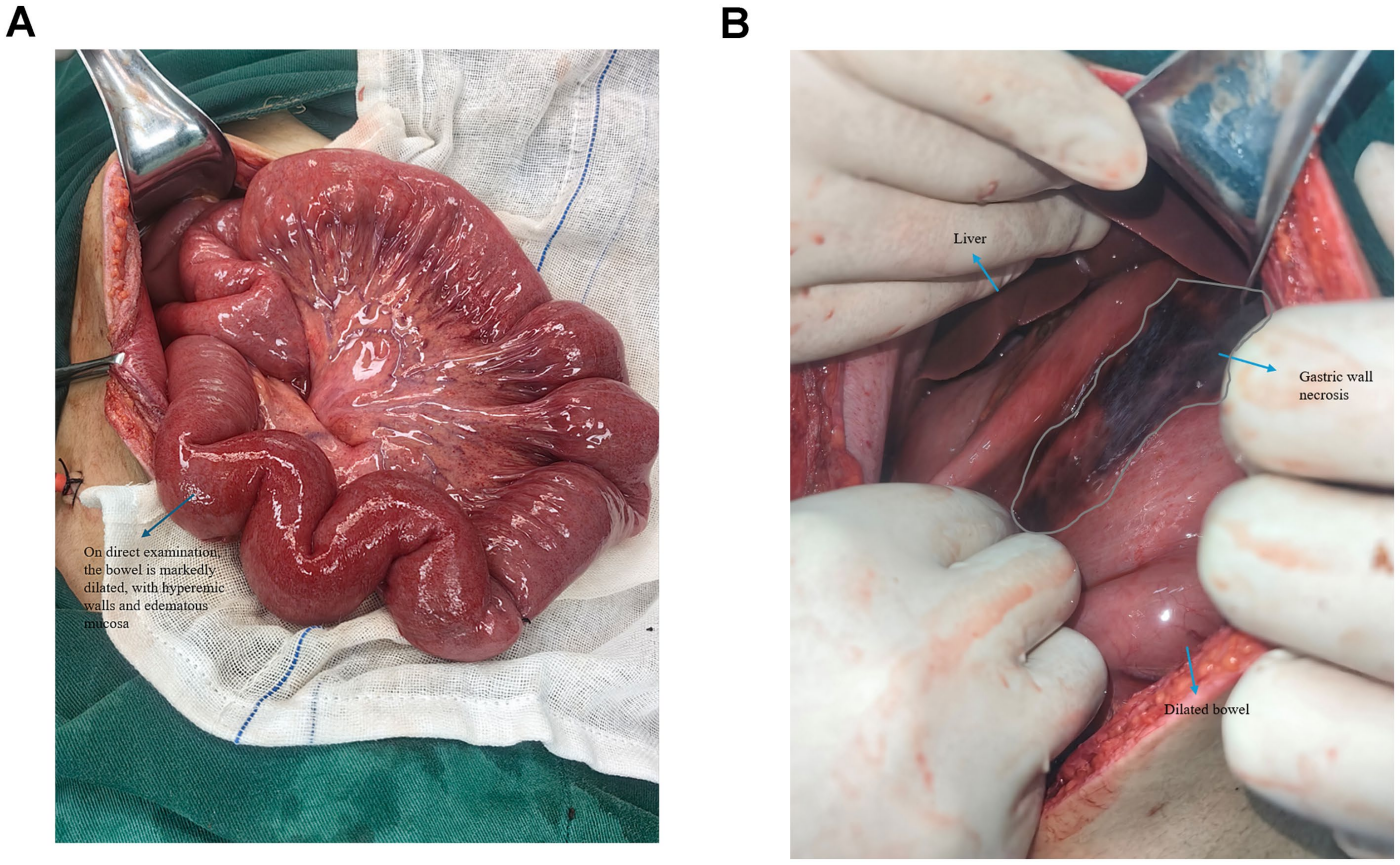
**(A,B)** During the operation, it was discovered that the small intestine was dilated and the gastric wall at the lesser curvature of the stomach had necrotized.

A nasogastric tube was retained, and emergency exploratory laparotomy was performed. The gastric body was found to be extensively dilated, with the lower margin extending into the pelvic cavity. A large volume of contents was present, and decompression yielded a significant amount of coffee-ground-like fluid. Examination revealed approximately 500 milliliters of pale red bloody fluid in the liver region, around the spleen, and within the intestinal spaces. No significant perforation, hemorrhage, or necrosis was observed in the small intestine, colon, or appendix. Extensive hemorrhagic necrosis measuring approximately 4 cm × 5 cm was noted on the lesser curvature of the anterior gastric wall near the cardia. A patchy necrotic focus measuring approximately 3 cm × 4 cm was identified on the greater curvature of the posterior gastric wall near the pylorus (Figures 2A,B). After adequate gastrointestinal decompression, the necrotic areas on the lesser curvature of the anterior gastric wall near the cardia and on the greater curvature near the pylorus were closed with continuous sutures using 3-0 (Johnson & Johnson) absorbable barbed sutures. The necrotic areas were then encased with continuous submucosal sutures using 3–0 absorbable sutures. A fistula tube was inserted via a puncture approximately 4 cm proximal to the gastric antrum on the anterior wall of the stomach, with its distal end reaching the jejunum. The gastric puncture site was sutured and secured, and the proximal end of the fistula tube was brought out through an incision in the left abdominal wall and secured. During surgery, a combination of sodium chloride and hydroxyethyl starch was administered to maintain blood pressure above 90/60 mmHg. After transfer to the ICU, the patient developed symptoms of septic shock. After 3 days of treatment with norepinephrine and meropenem, the patient’s blood pressure stabilized. The patient was then treated with methoxamine and cefoperazone/sulbactam for 2 days. Once vital signs were stable, the patient was transferred to a general ward. The patient was discharged 14 days postoperatively in stable condition. Follow-up laboratory tests showed normal results. Two outpatient follow-up visits post-discharge revealed no recurrence.

## Discussion

Acute gastric distension (AGD) is a rare but serious clinical condition first described by S. E. Dupuy in 1833 (4). The exact causes remain unclear, but they can generally be classified into mechanical and non-mechanical causes. Mechanical causes include outlet obstruction disorders such as pyloric stenosis and superior mesenteric artery syndrome. Non-mechanical causes include eating disorders, such as anorexia and bulimia, as well as local infections and medication-related factors, with the latter often occurring after binge eating (5, 6). The patient in this case had no history of anorexia nervosa or binge eating, nor did he suffer from any mechanical obstruction. The incident was caused solely by a bet with a friend to see who could eat more; the massive amount of potatoes and the hard-to-digest, high-fiber meat led to this outcome, and the cause can be attributed to binge eating. Clinically, vomiting is a prominent symptom observed in over 90% of AGD cases (7). Patients with acute gastrointestinal distress caused by gastric torsion may be unable to vomit due to obstruction at the gastroesophageal junction (8).

Despite the stomach’s rich vascular supply, AGD may cause venous congestion in the gastric wall, thereby increasing intragastric pressure. Intragastric pressure within the range of 14–30 cm H₂O correlates with gastric ischemia and poor prognosis (8–10).

In certain circumstances, ischemic AGD may progress to gastric perforation, emphysematous gastritis, or abdominal compartment syndrome. Rupture and perforation may lead to more pronounced symptoms, including signs of acute peritonitis. Other complications of AGD include arrhythmias, aortic occlusion, superior mesenteric artery syndrome, and mesenteric ischemia. Symptoms are often nonspecific (11).

Physical examination may reveal abnormal distension of the upper abdomen and positive hydrothorax, but the more diagnostically valuable mega-gastric sinus syndrome is uncommon. Most cases present with physical signs consistent with diffuse peritonitis.

The diagnosis of AGD is primarily based on clinical presentation and imaging studies, such as abdominal X-rays and computed tomography (CT) scans, which typically reveal marked gastric distension accompanied by a large amount of gas or fluid. Once perforation has been ruled out, gastroscopy may also be used to assess the cause and severity of the condition (10, 12). However, a significant number of cases are only diagnosed through emergency exploratory laparotomy following CT scans revealing definitive signs of peritonitis.

Management approaches for AGD vary widely, ranging from conservative management to invasive surgery. NGT decompression is the preferred method for hemodynamically stable patients (8, 13). As gastric distension progresses, despite conservative treatment, and leads to signs of vascular damage—manifesting as gastric wall necrosis, abdominal compartment syndrome, and acute renal failure—emergency surgical intervention is required (14, 15). In addition, the detection of extraperitoneal gas, such as free intraperitoneal air, pneumoperitoneum, or portal vein gas (most commonly caused by intestinal mucosal damage, increased intestinal pressure, and sepsis resulting from gas-producing bacteria, with a very poor prognosis)—serves as a critical warning sign indicating the need for emergency exploratory laparotomy (16–19).

Patients presenting with gastric necrosis, perforation, compartment syndrome, or symptoms of shock may undergo partial or total gastrectomy, as appropriate, in conjunction with esophagogastroduodenostomy and tube feeding (8, 20). In this case, as soon as the CT scan revealed gastric distension with air in the portal vein, we performed exploratory laparotomy and gastrointestinal decompression. The entire process, from admission to surgery, took less than 2 hours. In addition to the patient’s young age, the rapid relief of intra-abdominal pressure was key to his survival. After decompression surgery, it is generally best to admit the patient to the ICU for close monitoring and supportive care, as subsequent challenges related to infection and nutrition are critical to the patient’s survival. Our patient remained in the ICU for 5 days, receiving potent antibiotics and nutritional support, which enabled a relatively rapid recovery. Additionally, attention must be paid to potential surgical complications, such as intestinal obstruction, puncture site ulcers, and poor wound healing.

## Conclusion

The etiology of AGD remains unclear, and due to the lack of definitive diagnostic criteria, it is frequently underdiagnosed or misdiagnosed. Early diagnosis of AGD is crucial for preventing serious complications such as gastric ischemia or necrosis. For patients who may have mental disorders or psychological illnesses, caution should be exercised when abdominal pain and vomiting occur. The primary reason this case was successfully treated was the timely surgical intervention. The patient underwent surgery within 2 hours of arriving at our hospital. Although no perforation occurred, the patient had already developed shock symptoms, which further underscores the direct correlation between treatment efficacy and time in this condition.

## Data Availability

The raw data supporting the conclusions of this article will be made available by the authors, without undue reservation.

## References

[1] Watters A Gibson D Dee E Mascolo M Mehler PS. Superior mesenteric artery syndrome in severe anorexia nervosa: a case series. Clin Case Reports. (2020) 8:185–9. doi: 10.1002/ccr3.2577, 31998513 PMC6982477

[2] Vettoretto N Viotti F Taglietti L Giovanetti M. Acute idiopathic gastric necrosis, perforation and shock. J Emerg Trauma Shock. (2010) 3:304. doi: 10.4103/0974-2700.66564, 20930990 PMC2938514

[3] Todd SR Marshall GT Tyroch AH. Acute gastric dilatation revisited. Am Surg. (2000) 66:709–10. doi: 10.1177/000313480006600801, 10966022

[4] Mishima T Kohara N Tajima Y Maeda J Inoue K Ohno T . Gastric rupture with necrosis following acute gastric dilatation: report of a case. Surg Today. (2012) 42:997–1000. doi: 10.1007/s00595-012-0162-4, 22411075

[5] Anderson K Watters A Dee E Mehler PS. Can we predict the development of acute gastric dilatation in patients with anorexia nervosa? J Eat Disord. (2023) 11:212. doi: 10.1186/s40337-023-00937-2, 38031186 PMC10688114

[6] Shaikh DH Jyala A Mehershahi S Sinha C Chilimuri S. Acute gastric dilatation: a cause for concern. Case Rep Gastroenterol. (2021) 15:171–7. doi: 10.1159/000512401, 33708066 PMC7923726

[7] Sahoo MR Kumar AT Jaiswal S Bhujabal SN. Acute dilatation, ischemia, and necrosis of stomach without perforation. Case Rep Surg. (2013) 2013:984594. doi: 10.1155/2013/984594, 24222883 PMC3814073

[8] Islam S Shah A Naraynsingh V Harnarayan P. Acute gastric dilatation with ischemia and perforation requiring emergency Total gastrectomy: a case of suspected abdominal compartment syndrome. Cureus. (2023) 15:e41265. doi: 10.7759/cureus.41265, 37529800 PMC10390345

[9] Loi CM Chen KH. Total gastric necrosis following massive gastric dilatation due to superior mesenteric artery syndrome. Asian J Surg. (2023) 46:2363–4. doi: 10.1016/j.asjsur.2022.12.008, 36550012

[10] Núñez J García-Angarita FJ Puerta A Muñoz P Sanjuanbenito A. Sleeve gastrectomy for idiopathic acute gastric dilatation with transmural necrosis. Ann R Coll Surg Engl. (2021) 103:e275–7. doi: 10.1308/rcsann.2020.7121, 34431688 PMC10335213

[11] Bathobakae L Bashir R Venero S Wilkinson T Yuridullah R Cavanagh Y . Acute gastric dilatation: a retrospective case series from a single institution. Case Rep Gastroenterol. (2024) 18:439–48. doi: 10.1159/000541516, 39474155 PMC11521523

[12] Maung H Buxey KN Studd C Ket S. Acute gastric dilatation in a bulimic patient. Gastrointest Endosc. (2017) 85:455–7. doi: 10.1016/j.gie.2016.03.015, 26975234

[13] Park KB Nho WY. Abdominal compartment syndrome caused by severe acute gastric distension in a patient with COVID-19: a case report. Medicine (Baltimore). (2023) 102:e34326. doi: 10.1097/MD.0000000000034326, 37443515 PMC10343875

[14] Huang Z Li C Tang G. Superior mesenteric artery syndrome with acute gastric dilatation caused by binge eating in an adolescent. Korean J Intern Med. (2022) 38:572–3. doi: 10.3904/kjim.2022.344, 37357605 PMC10338254

[15] Usui A Kawasumi Y Ishizuka Y Hosokai Y Ikeda T Saito H . A case report of postmortem radiography of acute, fatal abdominal distension after binge eating. Am J Forensic Med Pathol. (2016) 37:223–6. doi: 10.1097/PAF.0000000000000243, 27571171

[16] Franzoi M Karic M Gröchenig HP. Abdominal compartment syndrome after binge eating. Gastroenterology. (2023) 164:e13–5. doi: 10.1053/j.gastro.2022.12.002, 36509154

[17] Han YJ Roy S Siau A Majid A. Binge-eating and sodium bicarbonate: a potent combination for gastric rupture in adults-two case reports and a review of literature. J Eat Disord. (2022) 10:157. doi: 10.1186/s40337-022-00677-9, 36348449 PMC9643985

[18] Jano F Behr C Almajali F Moran V. Acute gastric dilatation complicated by necrosis and perforation following a binge eating episode. Cureus. (2022) 14:e31727. doi: 10.7759/cureus.31727, 36569719 PMC9771086

[19] Pan Y. Hepatic portal vein gas associated with intestinal ischemia and acute gastric dilatation: a case report. Ann Palliat Med. (2021) 10:7095–8. doi: 10.21037/apm-20-1764, 33691431

[20] Shaikh OH Reddy N Kumbhar US Suresh C. Acute massive gastric dilatation as a result of closed-loop obstruction of stomach: an unusual and rare phenomenon. BMJ Case Rep. (2020) 13:e235943. doi: 10.1136/bcr-2020-235943, 32878832 PMC7470494

